# Supplementary material for: Complexome profiling of the Chlamydomonas psb28 mutant reveals TEF5 as an early PSII assembly factor
Source: Plant Cell. 2025 Mar 17;37(6):koaf055. doi: 10.1093/plcell/koaf055 (PMC12164590; doi:10.1093/plcell/koaf055)
Supplement: koaf055_Supplementary_Data [file koaf055_supplementary_data.zip › psb28_interactive.zip!/plots/intensity/qualityPlots/peptides/PHT8_Cre16.g675300.t1.1.html]

### Cre16.g675300.t1.1

Trivial name: PHT8  
SeqCoverage [median]: 6.60  
Mass: 60.16 kDa  
  
 

| Sequence | MissedCleavage | Mass [Da] | wtIntensity | muIntensity | Score |
| --- | --- | --- | --- | --- | --- |
| LADQFGGK | 0 | 834.42 | 1355.00 | 1292.28 | 81.30 |
| QLPEDGAPVANTTATAVGK | 0 | 1838.94 | 0.00 | 1527.76 | 78.49 |
| SIDMDTVQSSR | 0 | 1237.56 | 1436.14 | 2803.21 | 142.00 |
|  |  |  | 2791.1 | 5623.3 |  |
